# Unexpected Genetic Diversity of Nostocales (Cyanobacteria) Isolated from the Phyllosphere of the Laurel Forests in the Canary Islands (Spain)

**DOI:** 10.3390/microorganisms12122625

**Published:** 2024-12-18

**Authors:** Nereida M. Rancel-Rodríguez, Nicole Sausen, Carolina P. Reyes, Antera Martel Quintana, Barbara Melkonian, Michael Melkonian

**Affiliations:** 1 Departamento de Botánica, Ecología y Fisiología Vegetal, Universidad de La Laguna, 38200 San Cristóbal De La Laguna, Spain; 2 Institute for Plant Sciences, Department of Biology, University of Cologne, Zülpicher Str. 47b, 50674 Cologne, Germany; 3 Instituto Universitario de Bio-Orgánica “Antonio González”, Universidad de La Laguna, 38206 San Cristóbal De La Laguna, Spain; cpreyes@ull.edu.es; 4 Banco Español de Algas, Instituto de Oceanografía y Cambio Global, IOCAG, Universidad de Las Palmas de Gran Canaria, 35214 Telde, Spain; amartel@marinebiotechnology.org; 5 Integrative Bioinformatics, Department of Plant Microbe Interactions, Max Planck Institute for Plant Breeding Research, 50829 Cologne, Germany; bmelkonian@mpipz.mpg.de

**Keywords:** biodiversity, clonal culture, epiphyllous, heterocyte-forming cyanobacteria, rRNA sequence comparison

## Abstract

A total of 96 strains of Nostocales (Cyanobacteria) were established from the phyllosphere of the laurel forests in the Canary Islands (Spain) and the Azores (Portugal) using enrichment media lacking combined nitrogen. The strains were characterized by light microscopy and SSU rRNA gene comparisons. Morphologically, most strains belonged to two different morphotypes, termed “*Nostoc*-type” and “*Tolypothrix*-type”. Molecular phylogenetic analysis of 527 SSU rRNA gene sequences of cyanobacteria (95 sequences established during this study plus 392 sequences from Nostocales and 40 sequences from non-heterocyte-forming cyanobacteria retrieved from the databases) revealed that none of the SSU rRNA gene sequences from the phyllosphere of the laurel forests was identical to a database sequence. In addition, the genetic diversity of the isolated strains was high, with 42 different genotypes (44% of the sequences) recognized. Among the new genotypes were also terrestrial members of the genus *Nodularia* as well as members of the genus *Brasilonema*. It is concluded that heterocyte-forming cyanobacteria represent a component of the phyllosphere that is still largely undersampled in subtropical/tropical forests.

## 1. Introduction

The Canary Islands belong to the biogeographic region of Macaronesia (together with the Azores, Madeira, Salvajes, and Cabo Verde). The laurel forest is one of the most characteristic ecosystems of Macaronesia [1]. Associated with the orographic cloud formation zone, with trees that can reach 30 m in height, it has a complex biogeographic history [2]. The laurel forest is largely confined to the semi-humid mid-elevations of the mountainous western Canary Islands, especially on windward slopes [3,4]. These altitudinal limits also correspond to the area of the so-called “sea of clouds”, formed by the thermal inversion of the predominant NE trade winds [5]. One of the most striking aspects of subtropical and tropical rain forests, including the laurel forest of the Canary Islands, is the amount and diversity of organisms living on the surface of leaves, comprising the phyllosphere. The phyllosphere is a dynamic and stressful environment, subjected to variations in temperature, humidity, and incidence of UV radiation [6,7]. Despite these restrictions, it harbors a diverse and largely understudied community of organisms (e.g., bacteria, fungi, algae, lichens, and bryophytes (e.g., [8,9,10])) that play an important role in nutrient cycling in forest ecosystems [11,12].

There have been a large number of studies on cryptogamic biota in the Canarian laurel forest focused on bryophytes, ferns, fungi, and lichens (e.g., [13,14,15,16,17,18]), but none on the biodiversity of microalgae.

In humid tropical regions, leaves are frequently colonized by microalgae, predominantly cyanobacteria. In tropical and subtropical forests, terrestrial cyanobacteria, along with other organisms, compose extensive biofilms, which grow on a wide variety of substrates such as wood, soil, and rocks [19,20,21,22]. Such biofilms play important roles in these ecosystems, especially as carbon sinks and sources of soil fertility, mainly due to nitrogen (N_2_) fixation of cyanobacteria [23,24,25,26,27]. Epiphyllous cyanobacteria have been reported from different tropical regions [28,29,30,31,32], and their morphological richness in this biome [21,33,34] has led to the description of several novel genera and species in recent years [35,36,37,38,39,40,41,42,43]. Although the diversity of cyanobacteria in the phyllosphere of tropical forests has been studied by culture-independent techniques [37,44], isolation is still crucial for characterizing unknown cyanobacteria. Here, we describe the isolation of 96 clonal strains of heterocyte-forming cyanobacteria (Nostocales) from the phyllosphere of laurel forests in the Canary Islands and Sao Jorge, the Azores. We sequenced the 16S rRNA gene of 95 strains and discovered an unexpected genetic diversity of Nostocales from the phyllosphere of the laurel forests.

## 2. Materials and Methods

### 2.1. Sampling and Enrichment

**Figure 1:**
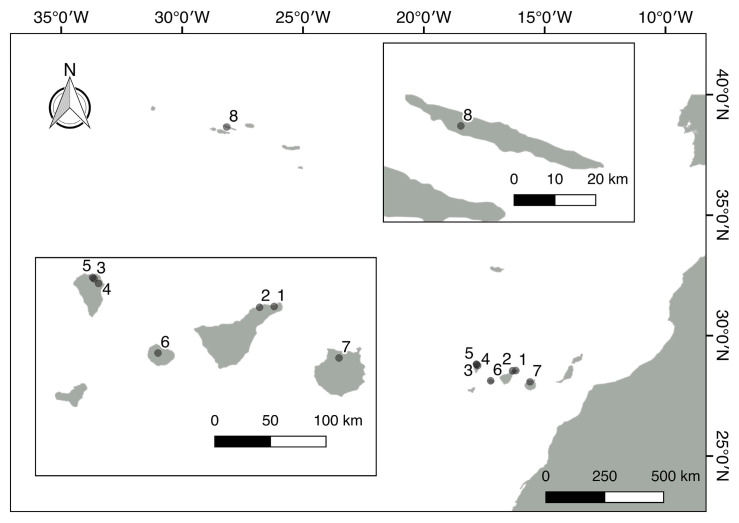
Sampling sites of laurel forests in the Canary Islands and Sao Jorge (the Azores). Numbers refer to sampling sites (localities) in Table 1.

This study was conducted in defined areas of the islands Tenerife, La Palma, La Gomera, and Gran Canaria (Figure 1); the main focus was on Tenerife and La Palma for the Canary Islands and Sao Jorge for the Azores (Table 1). Samples were collected randomly in different seasons (wet and dry seasons) throughout the sampling period.

**Figure 2:**
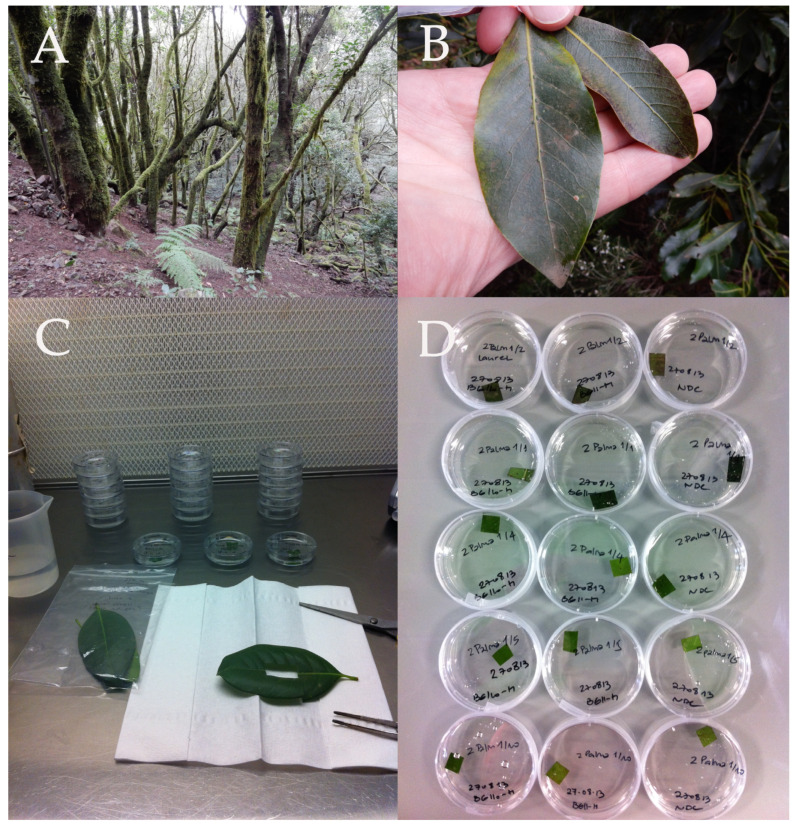
(**A**–**D**) Photographic documentation of sampling of leaves and set-up of enrichment cultures. (**A**) A typical view of the laurel forest of La Gomera (Canary Islands). The tree trunks are heavily colonized by epiphytes. (**B**) Two sampled leaves of different sizes from *Laurus novocanariensis*, with their upper surfaces partially covered by epiphylls. (**C**) Leaf material being prepared for enrichment cultures in a laminar flow hood. (**D**) Inoculated leaf disks in Petri dishes containing culture media for enrichment of heterocyte-forming cyanobacteria.

Leaves, representing the phyllosphere of the cyanobacteria to be isolated (Figure 2A–D), were collected mainly from members of Lauraceae (Juss), i.e., *Laurus novocanariensis*, *Appolonias barbujana*, *Ocotea foetens*, and *Laurus azorica*, and for comparison, leaves were collected from members of Aquifoliaceae (Bercht and J.Presl), i.e., *Ilex canariensis*, and Adoxaceae (E. Mey), i.e., *Viburnum rigidum*. A total of 215 samples were taken from a variety of potential habitats: the bark of laurel trees, soil and rain puddles close to the trees, bryophytes, wet walls, and particularly from leaves. Trees of different ages were probed. For each tree, young and mature leaves were cut and the leaf size and the position of the leaf on the tree (including the position on the branch and its distance from the ground) were recorded. Leaves with different surface appearances were collected: clean surfaces, rough surfaces, and surfaces covered with epiphylls.

For comparison, fallen leaves were collected, and additional samples were taken from the soil around the trees and the bark of the trees. All samples were kept separately in individual zip plastic bags. They were stored in a dry place at room temperature (20 °C) in darkness until they were used for the enrichment of heterocyte-forming cyanobacteria. The natural samples were processed approximately three months after exposing the leaf cuts to three different culture media (see below). After this time, the first heterocyte-forming cyanobacteria were detected in the enrichment samples.

### 2.2. Establishment of Clonal Cultures

Fresh leaves were treated under sterile conditions in a laminar flow bench (Figure 2A–D). Briefly, 1 cm^2^ pieces were cut under a stereo microscope (Nikon, SMZ1500, Tokyo, Japan). Each leaf cut was incubated in a Petri dish (60 mm in diameter) in one of three different culture media (BG11-H, BG11 0-H, and NDC; composition of media: Supplementary Tables S1 and S2). The enrichments were grown in an incubator under the following conditions: 25 °C, light intensity of 2–20 µmol photons m^−2^ s^−1^ (white LED tube Osram ST8V-1200 MM 16.2 W and daylight LED tube Osram 240713 ST8V-1200 MM 16.2 W; Osram, Munich, Germany), and a light/dark cycle of 14 h/10 h for a minimum of three months. A total of 179 leaves were collected, yielding 537 different enrichment cultures. The enrichment cultures were checked regularly (every 2 weeks) using an Olympus inverted microscope equipped with phase contrast optics (CK-41, Olympus, Hamburg, Germany). After the detection of heterocyte-forming cyanobacteria, the establishment of clonal cultures was initiated. Isolation of single cells, short filaments, or hormogonia was performed with a micropipette under the inverted microscope. Algae were transferred into 24-well microtiter plates (filled with 1 mL of BG11 0-H) and maintained in tubes/flasks in a growth chamber.

The cultures have been deposited in the Spanish Bank of Algae (Banco Español de Algas, https://marinebiotechnology.org/es/) and the Central Collection of Algal Cultures at the University of Duisburg-Essen, Germany (CCAC, https://www.uni-due.de/biology/ccac/). A list of strains and their strain numbers are provided in Supplementary Table S3.

### 2.3. Light Microscopical Analyses

Light microscopical investigations of isolated clones were performed with a Nikon 90i (Nikon Japan) microscope equipped with phase contrast optics. Quantitative evaluations were carried out with three-week-old cultures. Measurements were made on at least 20 different filaments.

### 2.4. DNA Extraction and Sequencing

DNA was extracted using a DNA extraction kit (Qiagen DNeasy plant mini kit, Qiagen GmbH, Hilden, Germany) according to the manufacturer’s instructions, with the following modifications: To harvest the cells, 1.5 mL of algal culture was centrifuged for 10 min at 10,000× *g* and the supernatant was discarded. To mechanically disrupt the cells, they were first frozen at −20 °C for at least one day. Sterile glass beads (diameter of 5 mm) were added to 20 μg of frozen pellets. All further steps were performed according to the manufacturer’s protocol until the elution step when DNA was eluted twice with 50 µL of heated elution buffer (65 °C) instead of 100 µL, which led to a significant increase in the final DNA concentration of the eluate. The DNA content was quantified via a NanoDrop 2000 spectrophotometer (Thermo Scientific; Rockford, IL, USA), and the DNA was stored at −20 °C until amplified using the Polymerase Chain Reaction (PCR) process as described in [45]. In this study, DNA was amplified with Hot Start PCR and High-Fidelity DNA Polymerase Thermo Scientific^®^ Phusion. DNA was amplified using a FlexCycler thermocycler (Analytik Jena, Jena, Germany). Fragment size and the amount of DNA can be estimated by comparison with a standard size marker. The sizes of the bands are about 1500 bp for the 16S rDNA region. Sequencing reactions were performed according to instructions of Macrogen Company (www.macrogen.com). In order to increase the sequenced region for each strain, four different primers were used per 16S region. The primers were designed based on DNA sequence alignments described in [46,47]. The sequences and names of the primers used for PCR amplification and sequencing of the 16S rDNA are shown in Table 2.

**Figure 3:**
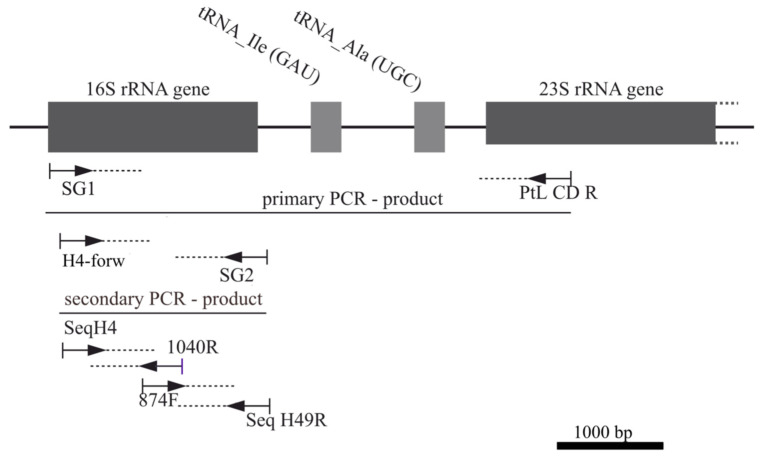
Overview of PCR and sequencing strategies for cyanobacterial 16S rRNA genes. The position of the primers used for amplification and sequencing of the 16S rRNA gene and the primary and secondary PCR products are shown. The general structure of the ribosomal rDNA operon with the 16S rRNA gene, two tRNA genes, and 23S rDNA gene is depicted schematically at the top of the figure. The primer sequences are shown in Table 2. Abbreviations used for the primers in the figure refer to the following primer designations and sequences in Table 2: 16S_SG1_short_forw (SG1), 16S H4_forw (H4-forw), ptLSU C-D_rev (PtL CD R), SG2_rev (SG2), Seq_16S_H4_forw (SeqH4), Seq_16S_1040_rev (1040R), Seq_16S_pos874_forw (874F), and Seq_16S_49_rev (Seq H49R).

The required concentration of primers was 5μM, and the minimum concentration of DNA was 50 ng/μL. At 4 °C, PCR components and primers 16S_SG1_short_forw and ptLSU C-D-rev were added for a primary PCR. As no detectable PCR products were achieved, 0.5–2 mL of the primary PCR was added to a second PCR with 16S H4_forw and the reverse primer SG2 (Figure 3).

The annealing temperature was calculated using an application associated with Phusion Polymerase (https://www.thermofisher.com/es/es/home/brands/thermo-scientific/molecular-biology/molecular-biology-learning-center/molecular-biology-resource-library/thermo-scientific-web-tools.html, accessed on 1 November 2024). Consensus sequences were read in Mesquite v3.02 (build 681) DNA sequence editor [48]. The newly sequenced 16S rDNA were aligned manually in SeaView 4.5.3 [49] as an alignment editor. rDNA operon alignments were constructed manually, guided by rRNA and tRNA secondary structures according to conserved rRNA secondary structure information obtained from the European Ribosomal RNA database (http://bioinformatics.psb.ugent.be/webtools/rRNA/).

### 2.5. Phylogenetic Analyses

The SSU rDNA from 95 heterocyte-forming cyanobacterial strains varied in length between 1357 and 1448 nucleotides (Supplementary Table S3 with accession numbers). The 16S DNA alignment contained 527 cyanobacterial sequences, of which 95 are new sequences of heterocyte-forming cyanobacteria from the laurel forest; the other 392 heterocyte-forming cyanobacterial sequences and 40 non-heterocyte-forming cyanobacterial sequences were retrieved from the NIH genetic sequence database GenBank^®^ ([50], https://www.ncbi.nlm.nih.gov/genbank, accessed on 1 September 2022). Hypervariable gene regions were analyzed on “The Mfold Web Server” [51]. Prior to phylogenetic analyses, sequences were confirmed by BLAST searches (Basic Local Alignment Search Tool); [52]. Several datasets for phylogenetic analyses in due consideration of the BLAST search results were created. In all datasets, a manually adjusted mask was applied, excluding the positions/hypervariable regions that could not be unambiguously aligned. The 16S rDNA dataset, containing 527 sequences and 1448 positions, was analyzed with maximum likelihood (RAxML). The model GTR + I + Γ and the parameters were estimated by RAxML. ML analyses were performed using the PTHREADS version of RAxML 7.2.6 [53]. To determine the best tree topology, 100 distinct ML trees were computed, starting from 20 distinct randomized maximum parsimony starting trees. Trees were sampled every 100 generations, and the “burn-in” (1,500,000 generations) was determined by the convergence criterion. The statistical support for branches was calculated by bootstrapping with 1000 replicates using ML [54]. Bootstrap values (B) ≥ 50 are depicted on the tree branches. The RAxML tree was rooted with *Gloeobacter violaceus*, *Synechococcus* sp. JA 2-3B, and *Synechococcus* sp. JA 3 3 AB. Adobe Illustrator CS5 (Adobe Systems, München, Germany) was used to convert tree files and for graphical tree design. A 16S rDNA “genotype sequence” was defined as differing by at least 3 bases from all other sequences in the dataset. Given the accuracy of our sequencing strategy (error rate < 0.1%), we expected few, if not no sequencing errors to have affected the designation of genotypes.

## 3. Results

### 3.1. Isolation of Strains

From the 215 samples, a total of 102 strains (96 still surviving) of heterocyte-forming cyanobacteria were isolated from a total of 38 leaves, a bryophyte sample, a soil sample, a sample of the bark of *Laurus novocanariensis*, and leaves of aquatic plants (Supplementary Table S3). The nonaxenic clonal strains of the heterocyte-forming cyanobacteria from the phyllosphere of the Macaronesian laurel forest have been deposited in two public culture collections (see Section 2 and Supplementary Table S3) and are available for further study.

### 3.2. Light Microscopical Observations

**Figure 4:**
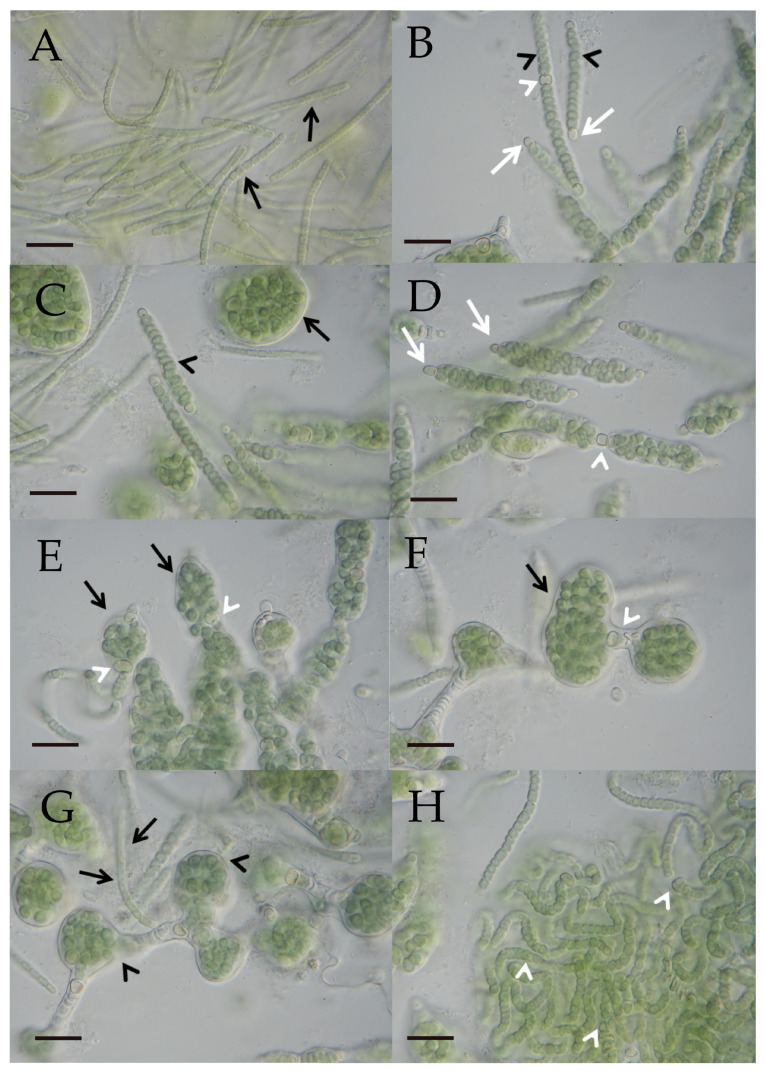
(**A**–**H**) Photographic documentation of the “*Nostoc*”-type morphotype (L066/CCAC 7008B/BEA 1768B) in a developmental sequence. (**A**) Several hormogonia (black arrows) characterized by barrel-shaped cells. Scale bar = 10 µm. (**B**) Differentiated filaments show lenticular to sublenticular vegetative cells (black arrowheads). Spherical to subspherical terminal heterocytes (white arrows) first appear on both ends of a filament, followed by a lenticular to sublenticular intercalary heterocyte (white arrowhead). Scale bar = 10 µm. (**C**) A common sheath develops surrounding the filament (black arrowhead). Within the sheath, vegetative cells continue to divide, expanding the sheath, with the filament eventually forming a globular structure (black arrow). Scale bar = 10 µm. (**D**) Terminal heterocytes (white arrows) and the initial intercalary heterocyte (white arrowhead) lack a sheath. Scale bar = 10 µm. (**E**,**F**) Curled filaments within expanded sheaths (black arrows) held together by intercalary heterocytes (white arrowheads). Scale bars = 10 µm. (**G**) When the flexible sheath (black arrowheads) breaks open, hormogonia (black arrows) emerge from the globular structures and start the developmental cycle again. Scale bar = 10 µm. (**H**) Filaments with intercalary heterocytes (white arrowheads) derived from a broken globular structure. Scale bar = 10 µm.

**Figure 5:**
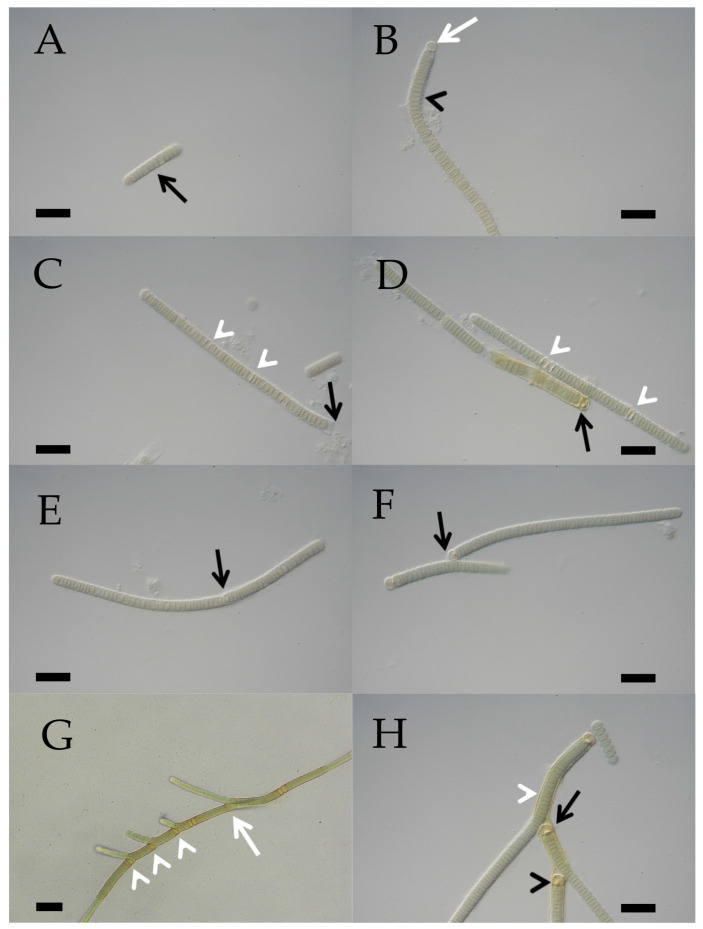
(**A**–**H**) Photographic documentation of the “*Tolypothrix*”-type morphotype (L088/CCAC 7034B/BEA 1790B) in a developmental sequence. (**A**) Hormogonia are characterized by lenticular to sublenticular cells (black arrow): note the slight polarity of the filament. Scale bar = 10 µm. (**B**) An older filament with lenticular to sublenticular vegetative cells (black arrowhead) and a spherical to subspherical terminal heterocyte (white arrow). Scale bar = 10 µm. (**C**) A firm sheath (black arrow) surrounds the straight filament. Early developmental stages of differentiation of intercalary heterocytes from vegetative cells (white arrowheads). Scale bar = 10 µm. (**D**) Lenticular to sublenticular differentiated intercalary heterocytes (white arrowheads; the left arrowhead depicts two adjacent intercalary heterocytes). The intercalary heterocytes remain enclosed in the firm sheath (unlike the situation in the “*Nostoc*”-type morphotype). A yellowish firm sheath surrounds vegetative cells of an older filament near a terminal heterocyte (black arrow). Scale bar = 10 µm. (**E**) Very early stage of the formation of a false branch. A vegetative cell adjacent to an intercalary heterocyte dissociates from the heterocyte and starts to bulge the sheath (black arrow). The intercalary heterocyte of the filament thus becomes a new terminal heterocyte. Scale bar = 10 µm. (**F**) A false branch attached to a heterocyte (black arrow). Scale bar = 10 µm. (**G**) Several false branches arising from vegetative cells adjacent to intercalary heterocytes (white arrowheads) or a necrotic cell (white arrow). Scale bar = 20 µm. (**H**) A primary (black arrow) and a secondary (black arrowhead) false branch share the same firm sheath (white arrowhead). Scale bar = 10 µm.

The morphology of heterocyte-forming cyanobacteria from 95 clonal cultures was investigated by light microscopy under identical controlled cultivation conditions. To stimulate heterocyte-formation, BG11 0-H (without combined nitrogen) was used for cultivation. For all strains, the development from single cells, short filaments, or hormogonia to mature filaments was documented. Images of all strains, highlighting the developmental sequence from hormogonia to mature filaments, have been presented in [55]. For the purpose of this study, it may be sufficient to illustrate only the two dominant morphotypes of Nostocales encountered in the strains isolated from the laurel forests, provisionally termed “*Nostoc*-type” (Figure 4A–H) and “*Tolypothrix*-type” (Figure 5A–H) here. Both represent adaptations to a terrestrial habitat and comprise the great majority of the heterocyte-forming cyanobacteria on leaves in the laurel forest: Among the 102 isolates, 53 strains belong to the “*Nostoc*-type, 36 belong to the “*Tolypothrix*”-type, and the remaining morphotypes belong to the genera *Nodularia*, *Brasilonema*, and *Scytonema*.

### 3.3. Molecular Phylogeny Using 16S DNA Gene Sequence Comparisons

**Figure 6:**
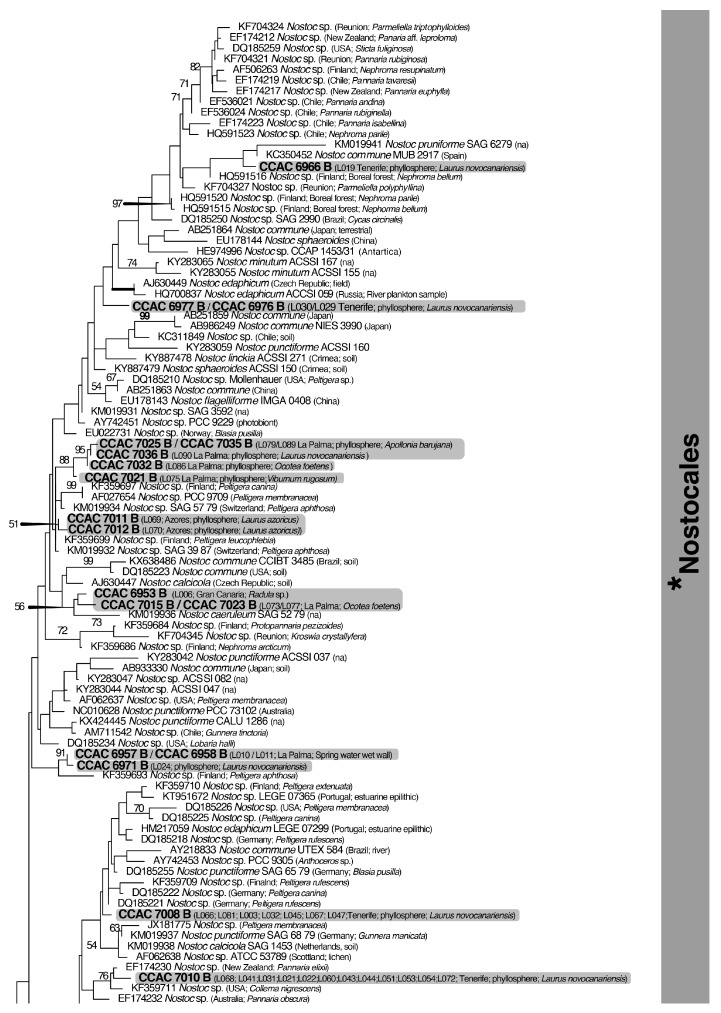

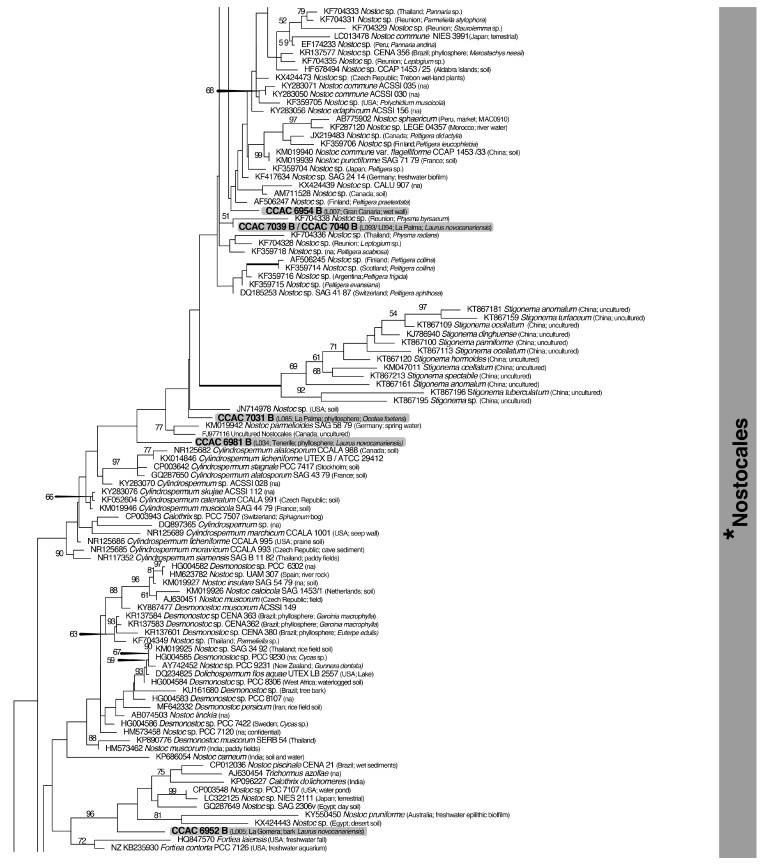

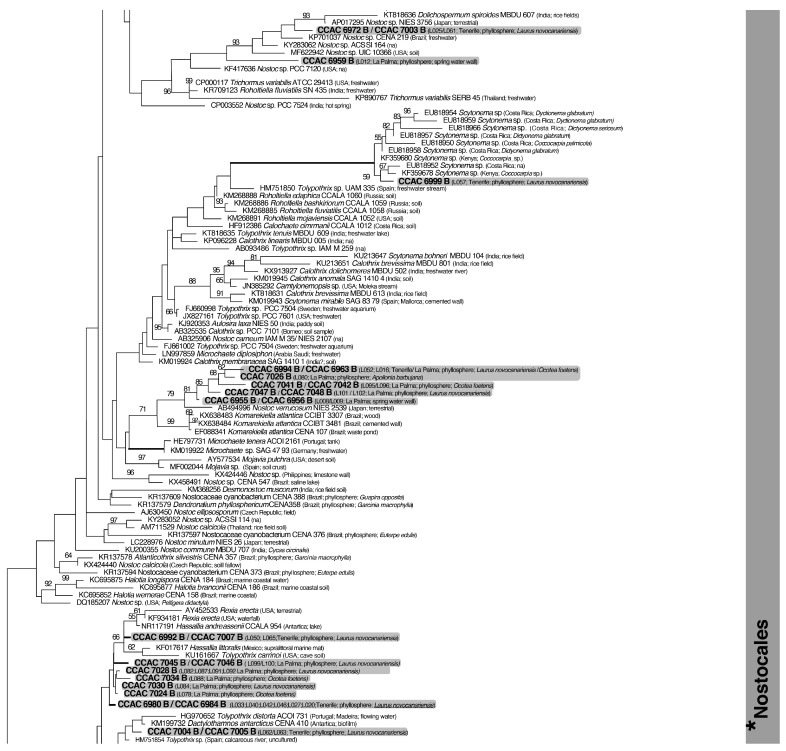

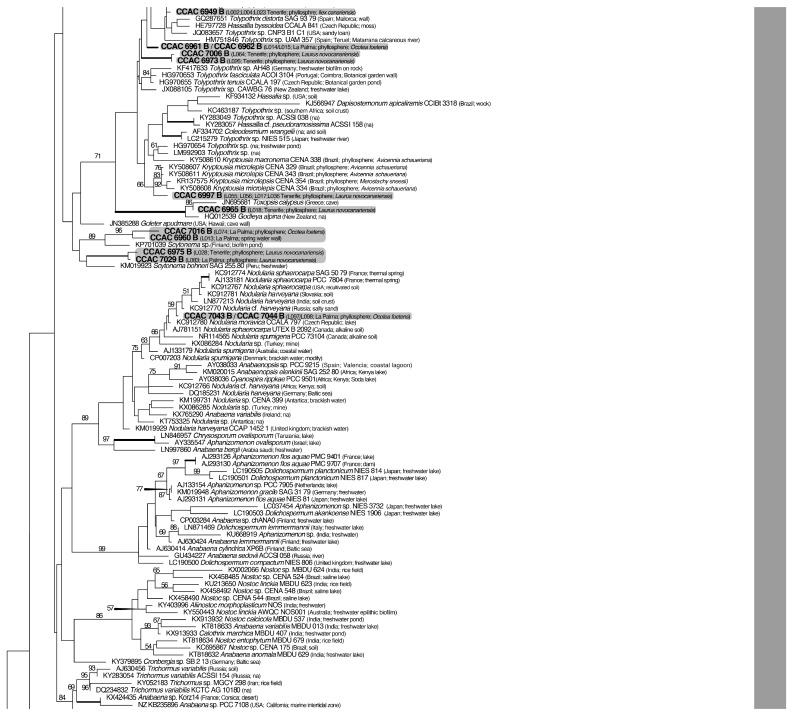

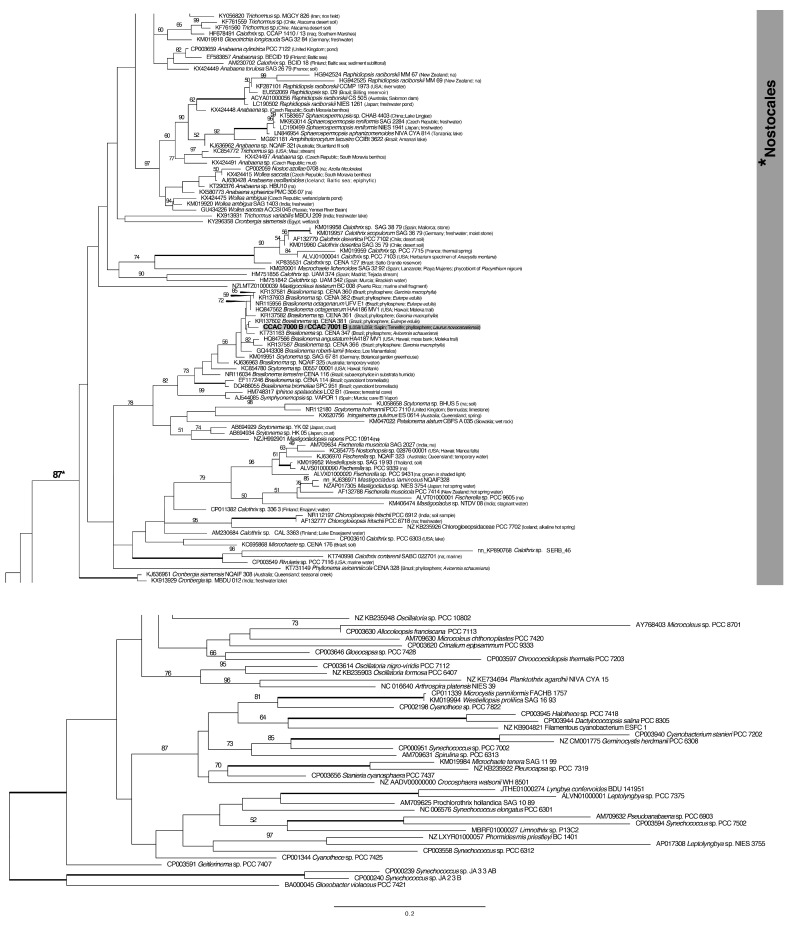
Phylogeny of heterocyte-forming cyanobacteria (Nostocales) from the phyllosphere of the laurel forests in the Canary Islands and the Azores using 16S rDNA sequence comparisons. The split tree (shown in 6 sections) was constructed with a database of 527 sequences, including 95 sequences generated during this study. The 16S rDNA dataset, with 1448 positions, was analyzed with maximum likelihood (RAxML). The model GTR + I + Γ and the parameters were estimated by RAxML (for further details, see Section 2). The sequences generated during this study are highlighted by a grey background and strain numbers (CCAC) in bold (strains with identical sequences are provided with their L numbers as well as the location and plant host in parentheses). Bootstrap values > 50% are shown; the bold value with an asterisk denotes support for the monophyly of the Nostocales. All tree branches in bold received maximal support. The complete merged tree is shown in Supplementary Figure S1.

One of the aims of this study was to determine the phylogenetic position of the heterocyte-forming cyanobacteria in the strain collection derived from the laurel forests. The goal of using this gene was to obtain first insights into the phylogenetic affiliation of the organisms under study and to evaluate their genetic diversity rather than to identify species since this would require additional markers. The final phylogenetic tree obtained by 16S rDNA sequence comparison is shown in Figure 6 (split tree; the merged tree is depicted in Supplementary Figure S1). As expected, the resolution obtained was limited, and many internal branches received no support. Heterocyte-forming cyanobacteria (Nostocales), however, represented a relatively well-supported (BV 87%) clade in this SSU rRNA phylogeny (Figure 6).

In total, 42 different genotypes could be distinguished (Figure 6). In the following section, the more prominent genotypes will be described briefly. The most abundant genotype is represented by 12 identical sequences derived from seven leaves of three plant species, four different sampling areas, and two islands (represented in the tree by CCAC 7010 B [L068] in Figure 6). Another prominent group of identical sequences, phylogenetically related to the previous group, is represented by seven strains from five leaves of two plant species, three sampling areas, and two islands (represented by CCAC 7008 B [L066] in Figure 6). Both groups of sequences belong to a larger assembly of sequences that include terrestrial species of genus *Nostoc* typically identified as *N. commune*, *N. punctiforme*, and *N. edaphicum* (Figure 6). Together with 19 further sequences representing 13 genotypes, these strains represent *Nostoc* s. str. [56]. The sequences comprised about 40% of the total sequences obtained in this study, and all strains exhibited the “*Nostoc*-type” morphotype (Figure 4A–H). Most of the database sequences in this assemblage were derived from symbiotic associations between *Nostoc* and fungi or plants. Isolates of *Nostoc* s. str. were obtained from the phyllosphere of six different plant species and were also recovered from a soil sample and a bryophyte sample. A second prominent group of sequences belonged to a moderately supported (BV 71%) clade (with a “*Tolypothrix*-type” phenotype; Figure 5A–H). It includes species mainly belonging to the genera *Tolypothrix*, *Hassallia*, and *Rexia* (Figure 6) and represents the family Tolypotrichaceae [56]. Together, isolates in Tolypotrichaceae represented 10 distinct genotypes (31 sequences) and about 33% of the total number of sequences obtained. The remaining 27% of the sequences, mostly of the “*Nostoc*-type” morphotype, could not be assigned to either of the two previously described assemblages/clades. Interestingly, four sequences represented strains that displayed neither a typical “*Nostoc*”- nor a “*Tolypothrix*-type” morphotype: The strains CCAC 7043 B (L097) and CCAC 7044 B (L098) were closely affiliated with the genus *Nodularia*, thus representing terrestrial members of this genus, and strains CCAC 7000 B (L058) and CCAC 7001 B (L059) belong to genus *Brasilonema* (Figure 6).

## 4. Discussion

The present report is the first systematic analysis of heterocyte-forming cyanobacteria (Nostocales) from the phyllosphere in the laurel forests of the Macaronesian islands. We chose an enrichment procedure using culture media lacking combined nitrogen to establish 96 clonal strains of heterocyte-forming cyanobacteria. No significant differences were observed regarding the development of heterocyte-forming cyanobacteria in the two culture media lacking combined nitrogen (BG11 0-H and NDC). Comparative light microscopical analyses of the strains identified two principal morphotypes, which we termed “*Nostoc*-type” and “*Tolypothrix*-type”. The first one is mostly equivalent to the genus *Nostoc* (s. str.), while the second one refers to species in Tolypotrichaceae. These represent the large majority of the strains isolated. In addition, we report the isolation of strains belonging to the genera *Nodularia* and *Brasilonema*. Surprisingly, none of the SSU rRNA gene sequences obtained for the 95 strains is identical to any database sequence, suggesting that the phyllosphere, especially in subtropical/tropical forests, is still largely undersampled with respect to heterocyte-forming cyanobacteria. The large number of unique genotypes recovered from the phyllosphere (42 out of 95 strains) supports this notion. It is clear that many of the new genotypes discovered during this study represent new taxa (genera or species) that need to be formally described, a process that has been initiated [57]. These findings regarding cyanobacteria from terrestrial habitats are not without precedent. Pham et al. [58] isolated 13 *Nostoc* strains from paddy soils in Vietnam and reported seven morphotypes and five genotypes associated with them. Similarly, Nguyen et al. [59] isolated 143 cyanobacterial strains of terrestrial cyanobacteria from man-made substrates on the campus of the University of the Ryukyus, Okinawa (Japan). They established 105 partial SSU rRNA gene sequences representing 30 generic types (including six types of Nostocales); almost all strains had no identical matches in GenBank. Sherwood et al. [60] collected 50 samples of cyanobacteria from Hawaiian freshwater and terrestrial habitats. Although many Hawaiian samples of cyanobacteria appeared to correspond to well-known genera morphologically, characterization with molecular techniques revealed that they are distinct taxa. Using a culture-independent approach, Rigonato et al. [44,61] reported a high genetic diversity of cyanobacteria (mostly Nostocales) from the phyllosphere in a Brazilian mangrove ecosystem and a Brazilian Atlantic forest. In consequence, in recent years, several studies have described new taxa (at the genus and species levels) of heterocyte-forming cyanobacteria from aerophytic/subaerophytic habitats in the pantropics (e.g., [35,36,38,39,41,42,43,62,63,64,65,66,67,68]). None of the newly described genera, except *Brasilonema* (see above), was represented in the isolated strains from the phyllosphere of the Macaronesian laurel forests (Figure 6). The two strains of *Brasilonema* (L058 and L059) were molecularly related to *B. octagenarum*, a species that causes serious damage to the leaves of *Eucalyptus* spp. [69,70]. Since the two strains of *Brasilonema* were isolated from the leaves of *Laurus novocanariensis*, a typical representative of the Macaronesian laurel forests, it is conceivable that extensive growth of *Brasilonema* spp. may also damage these leaves. However, given that we used an enrichment procedure to isolate Nostocales from the phyllosphere, we have no information on the extent of colonization of leaves by heterocyte-forming cyanobacteria or their ecological significance. Provisional analysis of laurel leaves using scanning electron microscopy failed to differentiate cyanobacterial filaments on the leaf surface from the other microorganisms present, such as fungi, bacteria, and green algae (*Phycopeltis* sp.; unpubl. observ.). This, as well as the long lag time (three months) encountered in enrichment cultures before heterocyte-forming cyanobacteria could be recognized, may indicate that dormant stages (i.e., akinetes) prevailed on the leaves, at least in the dry seasons. It is also possible that heterocyte-forming cyanobacteria on the laurel leaves are engaged in early lichenization processes, given that many isolates are closely related to genotypes known to be derived from lichenized Nostocales (Figure 6). Although we did not study the host specificity of the Nostocales in the phyllosphere in detail, it appears that both the “*Nostoc*”-type and the “*Tolypothrix*”-type strains displayed no host specificity, colonizing six and five out of seven tree species sampled, respectively. It is worth noting, however, that the “*Tolypothrix*”-type morphotypes seem to be confined to leaves, whereas the “*Nostoc*”-type morphotypes are distributed more widely, having been found on bark, among bryophytes, and on wet walls (Supplementary Material Table S3).

Cyanobacteria growing on leaf surfaces in tropical rainforests can fix atmospheric nitrogen [71]. Rates of fixation are strongly influenced by the presence of nutrients leached from the leaf as well as by light intensity, desiccation, and co-occurrence of epiphyllous lichens and bryophytes [12,23,24,29,72,73,74,75]. Cloud forests have been shown to lose more nitrogen in stream discharge than they gain from atmospheric deposition. In a study of epiphytes (bryophytes and lichens) that harbor N_2_-fixing cyanobacteria on *Quercus costaricensis* Liebm. in the Cordillera de Talamanca, Costa Rica, Markham and Otarola [76] determined the overall nitrogen input from N_2_-fixation to be 6.1 kg N ha^−1^ year^−1^ during the wet season, mostly in understory branches. These results show that tree epiphytes constitute a significant source of nitrogen for these forests, due to the trees’ large surface area, and can make up for nitrogen lost from these ecosystems. It has also recently been reported that asymbiotic nitrogen fixation in the phyllosphere can be significantly higher than in rhizospheric soil [27,75]. It is therefore conceivable that heterocyte-forming cyanobacteria, especially during the wet season, also play a significant role in balancing the nitrogen budget in the laurel forests of Macaronesia, a notion that deserves further study.

## Figures and Tables

**Table 1:** Sampling areas of leaves from laurel forests by island, along with their locality, geographic coordinates, altitude levels, and date of sampling.

Island	Locality	Geographic Coordinates and Altitude	Date of Sampling
Tenerife	Anaga National Park (**1**) *	28°32′57″ N–16°11′09″ W 727 m	17 October 2010 11 March 2012
	Pedro Álvarez forest (**2**) *	28°32′28″ N–16°18′65″ W 780 m	23 December 2012
La Palma	Los Tilos National Park (**3**) *	28°48′00″ N–17°49′00″ W	4 March 2011
	Cubo de la Galga (**4**) *	28°45′28″ N–17°46′34″ W	5 March 2011
	Barranco del Agua (**5**) *	28°48′46″ N–17°49′47″ W	14 August 2012
La Gomera	Garajonay National Park (**6**) *	28°8′3.73″ N–17°14′27.53″ W	3 December 2010 7 December 2013
Gran Canaria	Los Tilos de Moya (**7**) *	28°04′53″ N–15°35′53″ W	14 October 2010 17 November 2013
Sao Jorge	Parque Natural de Sao Jorge (the Azores) Reserva (**8**) *	38°40′13″ N–28°81′54″ W	18 April 2012

* Numbers in parentheses refer to sampling sites in Figure 1.

**Table 2:** PCR primers and sequencing primers used for amplification of the nuclear-encoded 16S rRNA gene (according to [46,47]).

**PCR Primer**	**Primer Sequence (5′ to 3′)**
16S_SG1_short_forw	GCAGAGAGTTYGATCCTGGCTCAGG
16S H4_forw	GATCCTKGCTCAGGATKAACGCTGGC
SG2_rev	CACGGATCCAAGGAGGTGATCCANCCNCACC
ptLSU C-D_rev	GCCGGCTCATTCTTCAAC
**Sequencing primer**	**Primer sequence (5′ to 3′)**
Seq_16S_H4_forw	TKGCTCAGGATKAACGCTGGC
Seq_16S_pos874_forw	ACTCAAAGGAATTGACG
Seq_16S_1040_rev	ACTTAACCCRACATCTCACGACACG
Seq_16S_49_rev	TACGGCTACCTTGTTACGACTTC

## Data Availability

Cyanobacterial strains used in this study are available from two public culture collections (CCAC and BEA, see Section 2 for affiliations of the collections and Supplementary Table S3 for strain numbers). The 16S rRNA sequences obtained during this study are deposited in GenBank.

## Supplementary Materials

The following supporting information can be downloaded at: https://www.mdpi.com/article/10.3390/microorganisms12122625/s1, Figure S1: 16S rRNA phylogeny of Nostocales (merged tree); Table S1: Composition of the BG 11 0-H culture medium; Table S2: Composition of the NDC culture medium; Table S3: List of isolated strains and strain numbers.

## References

[1] Morales D., Jimenez M.S., Gonzalez-Rodríguez A.M., Cermák J. (1996). Laurel forest in Tenerife, Canary Islands. I. The site, stand structure and stand leaf area distribution. Trees.

[2] Nogué S., De Nascimento L., Fernández-Palacios J.M., Whittaker R.J., Willis K.J. (2013). The ancient forests of La Gomera, Canary Islands, and their sensitivity to environmental change. J. Ecol..

[3] Fernández-Palacios J.M. (1992). Climatic responses of plant species on Tenerife, The Canary Islands. J. Veg. Sci..

[4] Del Arco Aguilar M.J., Rodriguez Delgado O., del Arco Aguilar M.J., Rodriguez Delgado O. (2018). Vegetation of the Canary Islands. Vegetation of the Canary Islands. Plant and Vegetation.

[5] Fernández–Palacios J.M., De Nicolás J.P. (1995). Altitudinal pattern of vegetation variation on Tenerife. J. Veg. Sci..

[6] Lindow S.E., Brandl M.T. (2003). Microbiology of the phyllosphere. Appl. Environ. Microbiol..

[7] Koskella B. (2020). The phyllosphere. Curr. Biol..

[8] Lambais M.R., Crowley D.E., Cury J.C., Büll R.C., Rodrigues R.R. (2006). Bacterial diversity in tree canopies of the Atlantic forest. Science.

[9] Peñuelas J., Terradas J. (2014). The foliar microbiome. Trends Plant Sci..

[10] Liu B.W., Li S.Y., Zhu H., Liu G.X. (2023). Phyllosphere eukaryotic microalgal communities in rainforests: Drivers and diversity. Plant Divers..

[11] Coxson D.S., Nadkarni N.M., Lowman M.D., Nadkarni N.M. (1995). Ecological roles of epiphytes in nutrient cycles of forest ecosystems. Forest Canopies.

[12] Zhu Y.G., Peng J., Chen C., Xiong C., Li S., Ge A., Wang E., Liesack W. (2023). Harnessing biological nitrogen fixation in plant leaves. Trends Plant Sci..

[13] González-González R., Léon M.C., Del Arco M.J. (2002). Los helechos de la Reserva Natural Integral de El Pijaral.

[14] Beltrán-Tejera E. (2006). Hongos, líquenes y briófitos del Parque Nacional de Garajonay (La Gomera, Islas Canarias).

[15] Patiño J., González-Mancebo J.M. (2010). Exploring the effect of host tree identity on epiphyte bryophyte communities in different Canarian subtropical cloud forests. Plant Ecol..

[16] Hernández-Hernández R., Castro J., Arco-Aguilar D., Fernández-López Á., González-Mancebo J.M. (2017). Post-fire salvage logging imposes a new disturbance that retards succession: The case of bryophyte communities in a Macaronesian laurel forest. Forests.

[17] González-Montelongo C., Pérez-Vargas I. (2019). Looking for a home: Exploring the potential of epiphytic lichens to colonize tree plantations in a Macaronesian laurel forest. For. Ecol. Manag..

[18] González-Montelongo C., Pérez-Vargas I. (2021). Is an invasive alien tree able to sustain a similar lichen diversity as the native forest? The case of the sweet chestnut (*Castanea sativa* Mill.) and the laurel forest in Macaronesia. For. Ecol. Manag..

[19] Büdel B., Weber H.M., Porembski S., Barthlott W. (2002). Cyanobacteria of inselbergs in the Atlantic rainforest zone of eastern Brazil. Phycologia.

[20] Gorbushina A.A. (2007). Life on the rocks. Environ. Microbiol..

[21] Sant’Anna C.L., Kaštovský J., Hentschke G.S., Komárek J. (2013). Phenotypic studies on terrestrial stigonematacean cyanobacteria from the Atlantic Rainforest, Sao Paulo State, Brazil. Phytotaxa.

[22] Glime J.M., Pócs T., Glime J.M. (2018). Chapter 8–6: Tropics: Epiphylls. Bryophyte Ecology.

[23] Freiberg E. (1998). Microclimatic parameters influencing nitrogen fixation in the phyllosphere in a Costa Rican premontane rain forest. Oecologia.

[24] Vitousek P.M., Cassman K.E.N., Cleveland C., Crews T., Field C.B., Grimm N.B., Howarth R.W., Marino R., Martinelli L., Rastetter E.B. (2002). Towards an ecological understanding of biological nitrogen fixation. Biogeochemistry.

[25] Bernap J., Prasse R., Harper K., Belnap J., Lange O.L. (2003). Influence of biological soil crusts on soil environments and vascular plants. Biological Soil Crusts: Structure, Function, and Management.

[26] Fürnkranz M., Wanek W., Richter A., Abell G., Rasche F., Sessitsch A. (2008). Nitrogen fixation by phyllosphere bacteria associated with higher plants and their colonizing epiphytes of a tropical lowland rainforest of Costa Rica. ISME J..

[27] Moreira J.C.F., Brum M., de Almeida L.C., Barrera-Berdugo S., de Souza A.A., de Camargo P.B., Oliveira R.S., Alves L.F., Pimentel Rosado B.H., Lambais M.R. (2021). Asymbiotic nitrogen fixation in the phyllosphere of the Amazon forest: Changing nitrogen cycle paradigms. Sci. Total Environ..

[28] Harrelson M.A. (1969). Tropical Epiphyllae and Nitrogen Fixation. Ph.D. Thesis.

[29] Bentley B.L. (1987). Nitrogen fixation by epiphylls in a tropical rainforest. Ann. Mo. Bot. Gard..

[30] Toomey M., Roberts D., Nelson B. (2009). The influence of epiphylls on remote sensing of humid forests. Remote Sens. Environ..

[31] Lemes-Da-Silva N.M., Branco L.H.Z., Necchi-Júnior O. (2010). New aerophytic morphospecies of Cyanobacteria from tropical forest fragments in northwestern São Paulo state, Brazil. Acta Bot. Brasil.

[32] Alvarenga D.O., Rigonato J., Branco L.H.Z., Fiore M.F. (2015). Cyanobacteria in mangrove ecosystems. Biodivers. Conserv..

[33] Branco L.H.Z., Hoffmann L., Teixeira J.P., Ferreira V., De Morais Filho J.C. (2009). Aerophytic cyanoprokaryotes from the Atlantic rainforest region of São Paulo State, Brazil: Chroococcales and Oscillatoriales. Cryptogam. Algol..

[34] Gama W.A., Laughinghouse H.D., Sant’Anna C.L. (2014). How diverse are coccoid cyanobacteria? A case study of terrestrial habitats from the Atlantic Rainforest (São Paulo, Brazil). Phytotaxa.

[35] Fiore M.F., Sant’Anna C.L., de Palva Azevedo M.T., Komárek J., Kaštovský J., Sulek J., Lorenzi A.S. (2007). The cyanobacterial genus *Brasilonema*, gen. nov., a molecular and phenotypic evaluation. J. Phycol..

[36] Sant’Anna C.L., de Palva Azevedo T.M., Kaštovský J., Komárek J. (2010). Two form-genera of aerophytic heterocytous cyanobacteria from Brasilian rainy forest “Mata Atlântica”. Fottea.

[37] Rigonato J., Alvarenga D.O., Andreote F.D., Dias A.C.F., Melo I.S., Kent A., Fiore M.F. (2012). Cyanobacterial diversity in the phyllosphere of a mangrove forest. FEMS Microbiol. Ecol..

[38] Hentschke G.S., Johansen J.R., Pietrasiak N., Fiore M.F., Rigonato J., Sant’Anna C.L., Komárek J. (2016). Phylogenetic placement of *Dapisostemon* gen. nov. and *Streptostemon*, two tropical heterocytous genera (Cyanobacteria). Phytotaxa.

[39] Johansen J.R., Mareš J., Pietrasiak N., Bohunická M., Zima J., Štenclová L., Hauer T. (2017). Highly divergent 16S rRNA sequences in ribosomal operons of *Scytonema hyalinum* (Cyanobacteria). PLoS ONE.

[40] Alvarenga D.O., Rigonato J., Branco L.H.Z., Melo I.S., Fiore M.F. (2016). *Phyllonema aviceniicola* gen. nov., sp. nov. and *Foliisarcina bertiogensis* gen. nov., sp. nov., epiphyllic cyanobacteria associated with *Avicennia schaueriana* leaves. Int. J. Syst. Evol. Microbiol..

[41] Alvarenga D.O., Andreote A.P.D., Branco L.H.Z., Fiore M.F. (2017). *Kryptousia macronema* gen. nov., sp. nov. and *Kryptousia microlepis* sp. nov., nostocalean cyanobacteria isolated from phyllospheres. Int. J. Syst. Evol. Microbiol..

[42] Bohunická M., Johansen J.R., Villaneuva C.D., Mareš M., Štenclová L., Becerra-Absalòn I., Hauer T., Kaštovský J. (2024). Revision of the pantropical genus *Brasilonema* (Nostocales, Cyanobacteria), with the description of 24 species new to science. Fottea.

[43] Hentschke G.S., De Souza Santos K.R., De Mattos L., Oliveira F., Vasconcelos V.M. (2024). A journey through Cyanobacteria in Brazil: A review of novel genera and 16S rRNA sequences. Cryptogam. Algol..

[44] Rigonato J., Gama W.A., Alvarenga D.O., Branco L.H.Z., Brandini F.P., Genuario D.B., FIORE M.F. (2016). *Aliterella atlantica* gen. nov., sp. nov., and *Aliterella antarctica* sp. nov., novel members of coccoid Cyanobacteria. Int. J. Syst. Evol. Microbiol..

[45] Saiki R.K., Gelfand D.H., Stoffel S., Scharf S.J., Higuchi R., Horn G.T., Mullis K.B., Erlich H.A. (1988). Primer-directed enzymatic amplification of DNA with a thermostable DNA polymerase. Science.

[46] Marin B., Nowack E.C., Melkonian M. (2005). A plastid in the making: Evidence for a second primary endosymbiosis. Protist.

[47] Marin B., Melkonian M. (2010). Molecular phylogeny and classification of the Mamiellophyceae class. nov. (Chlorophyta) based on sequence comparisons of the nuclear-and plastid-encoded rRNA operons. Protist.

[48] Maddison W.P. (2011). Mesquite: A Modular System for Evolutionary Analysis Version 2.75. http://mesquiteproject.org.

[49] Gouy M., Guindon S., Gascuel O. (2010). SeaView version 4: A multiplatform graphical user interface for sequence alignment and phylogenetic tree building. Mol. Biol. Evol..

[50] Sayers E.W., Cavanaugh M., Clark K., Pruitt K.D., Schoch C.L., Sherry S.T., Karsch-Mizrachi I. (2020). GenBank. Nucleic Acids Res..

[51] Zuker M. (2003). Mfold web server for nucleic acid folding and hybridization prediction. Nucleic Acids Res..

[52] Altschul S.F., Gish W., Miller W., Myers E.W., Lipman D.J. (1990). Basic local alignment search tool. J. Mol. Biol..

[53] Stamatakis A. (2014). RAxML version 8: A tool for phylogenetic analysis and post-analysis of large phylogenies. Bioinformatics.

[54] Felsenstein J. (1985). Confidence limits on phylogenies: An approach using the bootstrap. Evolution.

[55] Rancel Rodríguez N.M. (2016). Biodiversity of Epiphyllous Heterocyst-forming Cyanobacteria in the Laurel Forests of the Canary Islands. Ph.D. Thesis.

[56] Struncecký O., Ivanova A.P., Mareš J. (2023). An updated classification of cyanobacterial orders and families based on phylogenomic and polyphasic analysis. J. Phycol..

[57] Rancel-Rodríguez N.M., Vieira C., Sansón M. (2024). Terrestrial aerophytic cyanobacteria in the Canary Island laurel-forest (Laurisilva): Discovery of *Brasilonema novocanariensis* sp. nov. and *Rhizonema melkonianarum* sp. nov. from the *Laurus phyllosphere*. Diversity.

[58] Pham H.T., Nguyen L.T., Duong T.A., Bui D.T., Doan Q.T., Nguyen H.T., Mundt S. (2017). Diversity and bioactivities of nostocacean cyanobacteria isolated from paddy soil in Vietnam. Syst. Appl. Microbiol..

[59] Nguyen X.H., Sumimoto S., Suda S. (2017). Unexpected high diversity of terrestrial cyanobacteria from the campus of the University of the Ryukyus, Okinawa, Japan. Microorganisms.

[60] Sherwood A.R., Carlile A.L., Vaccarino M.A., Johansen J.R. (2015). Characterization of Hawaiian freshwater and terrestrial cyanobacteria reveals high diversity and numerous putative endemics. Phycol. Res..

[61] Rigonato J., Kent A.D., Alvarenga D.O., Andreote F.D., Beirigo R.M., Vidal-Torrado P., Fiore M.F. (2013). Drivers of cyanobacterial diversity and community composition in mangrove soils in south-east Brazil. Environ. Microbiol..

[62] Sant’Anna C.L., Azevedo M.P., Fiore M.F., Lorenzi A.S., Kaštovský J., Komárek J. (2011). Diversidade subgenérica de *Brasilonema* (Cyanobacteria, Scytonemataceae). Braz. J. Bot..

[63] Hauer T., Bohunická M., Muehlsteinova R. (2013). *Calochaete* gen. nov. (Cyanobacteria, Nostocales) a new cyanobacterial type from the “páramo” zone in Costa Rica. Phytotaxa.

[64] Hentschke G.S., Rigonato J., Genuário D.B., Laughinghouse H.D., Sant’Anna C.L. (2019). Morphological and molecular characterization of *Stigonema jureiensis* sp. nov.(Nostocales, Cyanobacteria) from the Atlantic Rainforest, São Paulo, Brazil. Fottea.

[65] Villanueva C.D., Garvey A.D., Hašler P., Dvořák P., Poulíčková A., Norwich A.R., Casamatta D.A. (2019). Descriptions of *Brasilonema geniculatum* and *Calothrix dumus* (Nostocales, Cyanobacteria): Two new taxa isolated from cemetery tombstones. Phytotaxa.

[66] Cai F., Li R. (2020). *Purpureonostoc*, a new name for a recently described genus of *Nostoc*-like cyanobacteria. Fottea.

[67] Mishra D., Saraf A., Kumar N., Pal S., Singh P. (2021). Issues in cyanobacterial taxonomy: Comprehensive case study of unbranched, false branched and true branched heterocytous cyanobacteria. FEMS Microbiol. Lett..

[68] Kaštovský J. (2024). Welcome to the jungle!: An overview of modern taxonomy of cyanobacteria. Hydrobiologia.

[69] Aguiar R., Fiore M.F., Franco M.W., Ventrella M.C., Lorenzi A.S., Vanetti C.A., Alfenas A.C. (2008). A novel epiphytic cyanobacterial species from the genus *Brasilonema* causing damage to *Eucalyptus* leaves. J. Phycol..

[70] Alvarenga D.O., Franco M.W., Sivonen K., Fiore M.F., Varani A.M. (2020). Evaluating *Eucalyptus* leaf colonization by *Brasilonema octagenarum* (Cyanobacteria, Scytonemataceae) using in planta experiments and genomics. PeerJ.

[71] Bentley E.L., Carpenter E.J. (1980). Effects of desiccation and rehydration on nitrogen fixation by epiphylls in a tropical rainforest. Microb. Ecol..

[72] Freiberg E. (1999). Influence of microclimate on the occurrence of cyanobacteria in the phyllosphere in a premontane rain forest of Costa Rica. Plant Biol..

[73] Cleveland C.C., Townsend A.R., Schimel D.S., Fisher H., Howarth R.W., Hedin L.O., Perakis S.S., Latty E.F., von Fischer J.C., Elseroad A. (1999). Global patterns of terrestrial biological nitrogen (N_2_) fixation in natural ecosystems. Glob. Biogeochem. Cycl..

[74] Pons J., Barraclough T.G., Gomez-Zurita J., Cardoso A., Duran D.P., Hazell S., Kamoun S., Sumlin W.D., Vogler A.P. (2006). Sequence-based species delimitation for the DNA taxonomy of undescribed insects. Syst. Biol..

[75] Alvarenga D.O., Clasen L.A., Thomsen A.M.R., Andersen R.F., Rousk K. (2024). Light drives nitrogen fixation in tropical montane cloud forests in Costa Rica. Sci. Total Environ..

[76] Markham J., Otárola M.F. (2021). Bryophyte and lichen biomass and nitrogen fixation in a high elevation cloud forest in Cerro de La Muerte, Costa Rica. Oecologia.

