# Supplementary material for: Unexpected Genetic Diversity of Nostocales (Cyanobacteria) Isolated from the Phyllosphere of the Laurel Forests in the Canary Islands (Spain)
Source: Microorganisms. 2024 Dec 18;12(12):2625. doi: 10.3390/microorganisms12122625 (PMC11676812; doi:10.3390/microorganisms12122625)
Supplement: Supplementary file 1 [file microorganisms-12-02625-s001.zip › microorganisms-3324672-supplementary.pdf]

Figure S1. 16S rRNA phylogeny of Nostocales, merged tree

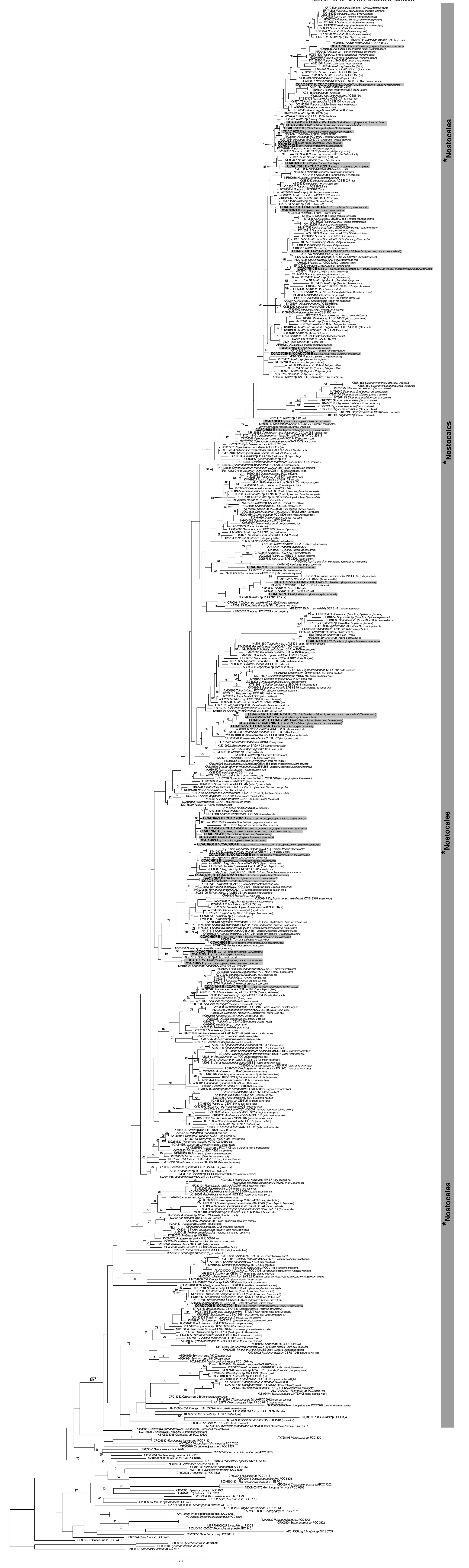

Table S1. Composition of the BG 11 0-H culture medium used in this study

(BG11-H: Modified by Naumann 2004 (unpublished). BG11: Stanier R. Y., Kunisawa R., Mandel M., Cohen-Bazire G. 1971; Purification and properties of unicellular blue-green algae (order Chroococcales). Bacteriological Reviews 35:171–205.

	**BG11-H (pH 7.3-7.4)**		**BG11 0-H (pH 7.3-7.4)**		
	Volume 1L	Molarity	Volume 1L	Molarity	
					**Stock 1L**
**Stock I (Main elements)**					40mL
$NaNO_3$	37.5 g	17.65mM	-	-	
K_2HPO4	1.0g	0.18mM	1.0g	0.18mM	
MgSO4 x 7 H_2O	1.875g	0.30mM	1.875g	0.30mM	
$CaCl_2$ x 2 H_2O	1.620g	0.25mM	1.620g	0.25mM	
Na_2 CO_3	0.5g	0.19mM	0.5g	0.19mM	
**Stock II (Trace Metals)**					1mL
H_3BO_3	2.86g	46.26µM	2.86g	46.26µM	
$MnCl_2$ x 4H2O	1.81g	9.15µM	1.81g	9.15µM	
$ZnSO_4$ x 7 H_2O	0.22g	0.77µM	0.22g	0.77µM	
Na_2MoO_4 x 2 H_2O	0.39g	1.6µM	0.39g	1.6µM	
$CuSO_4$ x 5 H_2O	79.00mg	0.32µM	79.00mg	0.32µM	
$Co(NO_3)_2$ x 6 H_2O	49.40mg	0.17µM	49.40mg	0.17µM	
**Stock III (Fe EDTA**					1mL

EDTA (Titriplex II)	5.22g	8.90µM	5.22g	8.90µM	
$FeSO_4$ x $7H_2O$	4.98g		4.98g		
KOH	54.00mL	1.00N	54,.0mL	1.00N	
**Stock IV (Vitamins)**					1mL
Vitamin B_{12}	0.20mg	0.15nM	0.20mg	0.15nM	
Biotin	1.00mg	4.00nM	1.00mg	4.00nM	
Thiamin-HCl	100.00mg	0.30µM	100.00mg	0.30µM	
Nicotinamide	0.10mg	0.80nM	0.10mg	0.80nM	
**Buffer**					1mL
HEPES	238.31g	1.00mM	238.31g	1.00mM	

Table S2. Composition of the NDC culture medium used in this study

NDC-Modified (NDC, Nitrogen-Deficient Cyanobacteria medium: Kusel-Fetzmann, E., Schagerl, M. 1992. Verzeichnis der Sammlung von Algen-Kulturen an der Abteilung für Hydrobotanik am Institut für Pflanzenphysiologie der Universität Wien. *Phyton*, 32 209-234.

**NDC-Modified**			
**#**	**Component**	**Final concentration**	**Addition per 1 L culture medium**
1	$MgSO_4 \cdot x\ 7H_2O$	1.00 mM	250 mg
2	$CaCl_2$ x $2H_2O$	0.75 mM	110 mg
3	NaCl	3.94 mM	230 mg
4	K_2HPO_4	1.56 mM	230 mg
5	$FeCl_3$ x· $6H_2O$	0.074 mM	20 mg
6	Trace metals*	See below	1 mL

***Preparation of Trace Metal Solution**
Na_2EDTA 4.36 g
$FeCl_3$ x 6 H_2O 3.15 g
in 1,000 mL MilliQ water, then add 1 mL of Primary Trace Metals**

****Primary Trace metals are stored frozen as 1 mL aliquots of a stock mixture (1-9).**		
#	Component	Addition per 100 mL MilliQ water
1	K_2CrO_4	0.194 g/100 mL
2	$CoCl_2$ x 6 H_2O	1.00 g/100 mL
3	$CuSO_4$ x 5 H_2O	0.25 g/100 mL
4	$MnCl_2$ x 4 H_2O	18.00 g/100 mL
4	Na_2MoO_4 x 2 H_2O	1.89 g/100 mL
6	$NiSO_4$ x 6 H_2O	0.27 g/100 mL
7	H_2SeO_3	0.13 g/100 mL

8	Na_3VO_4	0.184 g/100 mL
9	$ZnSO_4$ x 7 H_2O	2.20 g/100 mL

Table S3: List of isolated strains and strain numbers.

L number	Island	Sampling site	Sample	Tree sp.	CCAC strain number	BEA strain number	16SrDNA accession number
L 002	Tenerife	2	Leaf	*Ilex canariensis* Poir. In Lamarck	CCAC 6949 B	BEA 1704B	**PQ659164**
L 003	Tenerife	2	Leaf	*Ilex canariensis* Poir. In Lamarck	CCAC 6950 B	BEA 1705B	**PQ659143**
L 004	Tenerife	2	Leaf	*Ilex canariensis* Poir. In Lamarck	CCAC 6951 B	BEA 1706B	**PQ659164**
L 005	La Gomera	6	Bark	*Laurus novocanariensis* Rivas-Mart., Lousa, Fern. Prieto, E. Días, J.C. Costa & C. Aguiar	CCAC 6952 B	BEA 1707B	**PQ659149**
L 006	Gran Canaria	7	Bryophyte (*Radula* sp.)		CCAC 6953 B	BEA 1708B	**PQ659139**
L 007	Gran Canaria	7	Green spot on wet wall		CCAC 6954 B	BEA 1709B	**PQ659145**
L 008	La Palma	3	Leaf on the wall (Spring water)		CCAC 6955 B	BEA 1710B	**PQ659155**
L 009	La Palma	3	Leaf on the wall (Spring water)		CCAC 6956 B	BEA 1711B	**PQ659155**
L 010	La Palma	3	Leaf on the wall (Spring water)		CCAC 6957 B	BEA 1712B	**PQ659141**
L 011	La Palma	3	Leaf on the wall (Spring water)		CCAC 6958 B	BEA 1713B	**PQ659141**
L 012	La Palma	3	Leaf on the wall (Spring water)		CCAC 6959 B	BEA 1714B	**PQ659151**
L 013	La Palma	3	Leaf on the wall (Spring water)		CCAC 6960 B	BEA 1715B	**PQ659171**

L 014	La Palma	4	Leaf	*Ocotea foetens* (Aiton) Baill	CCAC 6961 B	BEA 1716B	**PQ659165**
L 015	La Palma	4	Leaf	*Ocotea foetens* (Aiton) Baill	CCAC 6962 B	BEA 1717B	**PQ659165**
L 016	La Palma	3	Leaf	*Ocotea foetens* (Aiton) Baill	CCAC 6963 B	BEA 1718B	**PQ659152**
L 017	Tenerife	1	Leaf	*Laurus novocanariensis* Rivas-Mart., Lousa, Fern. Prieto, E. Días, J.C. Costa & C. Aguiar	CCAC 6964 B	BEA 1719B	**PQ659168**
L 018	Tenerife	1	Leaf	*Laurus novocanariensis* Rivas-Mart., Lousa, Fern. Prieto, E. Días, J.C. Costa & C. Aguiar	CCAC 6965 B	BEA 1720B	**PQ659169**
L 019	Tenerife	1	Leaf	*Laurus novocanariensis* Rivas-Mart., Lousa, Fern. Prieto, E. Días, J.C. Costa & C. Aguiar	CCAC 6966 B	BEA 1721B	**PQ659131**
L 020	Tenerife	1	Leaf	*Laurus novocanariensis* Rivas-Mart., Lousa, Fern. Prieto, E. Días, J.C. Costa & C. Aguiar	CCAC 6967 B	BEA 1722B	**PQ659162**
L 021	Tenerife	1	Leaf	*Laurus novocanariensis* Rivas-Mart., Lousa, Fern. Prieto, E. Días, J.C. Costa & C. Aguiar	CCAC 6968 B	BEA 1723B	**PQ659144**
L 022	Tenerife	1	Leaf	*Laurus novocanariensis* Rivas-Mart., Lousa, Fern. Prieto, E. Días, J.C. Costa & C. Aguiar	CCAC 6969 B	BEA 1724B	**PQ659144**

L 023	Tenerife	1	Leaf	*Laurus novocanariensis* Rivas-Mart., Lousa, Fern. Prieto, E. Días, J.C. Costa & C. Aguiar	CCAC 6970 B	BEA 1725B	**PQ659164**
L 024	Tenerife	1	Leaf	*Laurus novocanariensis* Rivas-Mart., Lousa, Fern. Prieto, E. Días, J.C. Costa & C. Aguiar	CCAC 6971 B	BEA 1726B	**PQ659142**
L 025	Tenerife	1	Leaf	*Laurus novocanariensis* Rivas-Mart., Lousa, Fern. Prieto, E. Días, J.C. Costa & C. Aguiar	CCAC 6972 B	BEA 1727B	**PQ659150**
L 026	Tenerife	1	Leaf	*Laurus novocanariensis* Rivas-Mart., Lousa, Fern. Prieto, E. Días, J.C. Costa & C. Aguiar	CCAC 6973 B	BEA 1728B	**PQ659167**
L 027	Tenerife	1	Leaf	*Laurus novocanariensis* Rivas-Mart., Lousa, Fern. Prieto, E. Días, J.C. Costa & C. Aguiar	CCAC 6974 B	BEA 1729B	**PQ659162**
L 028	Tenerife	1	Leaf	*Laurus novocanariensis* Rivas-Mart., Lousa, Fern. Prieto, E. Días, J.C. Costa & C. Aguiar	CCAC 6975 B	BEA 1730B	**PQ659173**
L 029	Tenerife	1	Leaf	*Laurus novocanariensis* Rivas-Mart., Lousa, Fern. Prieto, E. Días, J.C. Costa & C. Aguiar	CCAC 6976 B	BEA 1731B	**PQ659132**

L 030	Tenerife	1	Leaf	*Laurus novocanariensis* Rivas-Mart., Lousa, Fern. Prieto, E. Días, J.C. Costa & C. Aguiar	CCAC 6977 B	BEA 1732B	**PQ659132**
L 031	Tenerife	1	Leaf	*Laurus novocanariensis* Rivas-Mart., Lousa, Fern. Prieto, E. Días, J.C. Costa & C. Aguiar	CCAC 6978 B	BEA 1733B	**PQ659144**
L 032	Tenerife	1	Leaf	*Laurus novocanariensis* Rivas-Mart., Lousa, Fern. Prieto, E. Días, J.C. Costa & C. Aguiar	CCAC 6979 B	BEA 1734B	**PQ659143**
L 033	Tenerife	1	Leaf	*Laurus novocanariensis* Rivas-Mart., Lousa, Fern. Prieto, E. Días, J.C. Costa & C. Aguiar	CCAC 6980 B	BEA 1735B	**PQ659162**
L 034	Tenerife	1	Leaf	*Laurus novocanariensis* Rivas-Mart., Lousa, Fern. Prieto, E. Días, J.C. Costa & C. Aguiar	CCAC 6981 B	BEA 1736B	**PQ659148**
L 035	Tenerife	1	Leaf	*Laurus novocanariensis* Rivas-Mart., Lousa, Fern. Prieto, E. Días, J.C. Costa & C. Aguiar	CCAC 6982 B	BEA 1737B	**PQ659173**
L 036	Tenerife	1	Leaf	*Laurus novocanariensis* Rivas-Mart., Lousa, Fern. Prieto, E. Días, J.C. Costa & C. Aguiar	CCAC 6983 B	BEA 1738B	**PQ659168**

L 040	Tenerife	1	Leaf	*Laurus novocanariensis* Rivas-Mart., Lousa, Fern. Prieto, E. Días, J.C. Costa & C. Aguiar	CCAC 6984 B	BEA 1742B	**PQ659162**
L 041	Tenerife	1	Leaf	*Laurus novocanariensis* Rivas-Mart., Lousa, Fern. Prieto, E. Días, J.C. Costa & C. Aguiar	CCAC 6985 B	BEA 1743B	**PQ659144**
L 042	Tenerife	1	Leaf	*Apollonia barujana* (Cav.) A. Braun	CCAC 6986 B	BEA 1744B	**PQ659162**
L 043	Tenerife	1	Leaf	*Apollonia barujana* (Cav.) A. Braun	CCAC 6987 B	BEA 1745B	**PQ659144**
L 044	Tenerife	1	Leaf	*Laurus novocanariensis* Rivas-Mart., Lousa, Fern. Prieto, E. Días, J.C. Costa & C. Aguiar	CCAC 6988 B	BEA 1746B	**PQ659144**
L 045	Tenerife	1	Leaf	*Laurus novocanariensis* Rivas-Mart., Lousa, Fern. Prieto, E. Días, J.C. Costa & C. Aguiar	CCAC 6989 B	BEA 1747B	**PQ659143**
L 046	Tenerife	1	Leaf	*Persea indica* (L.) Spreng	CCAC 6990 B	BEA 1748B	**PQ659162**
L 047	Tenerife	1	Leaf	*Laurus novocanariensis* Rivas-Mart., Lousa, Fern. Prieto, E. Días, J.C. Costa & C. Aguiar	CCAC 6991 B	BEA 1749B	**PQ659143**
L 050	Tenerife	1	Leaf	*Laurus novocanariensis* Rivas-Mart., Lousa, Fern. Prieto, E. Días, J.C. Costa & C. Aguiar	CCAC 6992 B	BEA 1904B	**PQ659156**

L 051	Tenerife	1	Leaf	*Laurus novocanariensis* Rivas-Mart., Lousa, Fern. Prieto, E. Días, J.C. Costa & C. Aguiar	CCAC 6993 B	BEA 1753B	**PQ659144**
L 052	Tenerife	1	Leaf	*Laurus novocanariensis* Rivas-Mart., Lousa, Fern. Prieto, E. Días, J.C. Costa & C. Aguiar	CCAC 6994 B	BEA 1754B	**PQ659152**
L 053	Tenerife	1	Leaf	*Laurus novocanariensis* Rivas-Mart., Lousa, Fern. Prieto, E. Días, J.C. Costa & C. Aguiar	CCAC 6995 B	BEA 1755B	**PQ659144**
L 054	Tenerife	1	Leaf	*Laurus novocanariensis* Rivas-Mart., Lousa, Fern. Prieto, E. Días, J.C. Costa & C. Aguiar	CCAC 6996 B	BEA 1756B	**PQ659144**
L 055	Tenerife	1	Leaf	*Laurus novocanariensis* Rivas-Mart., Lousa, Fern. Prieto, E. Días, J.C. Costa & C. Aguiar	CCAC 6997 B	BEA 1757B	**PQ659168**
L 056	Tenerife	1	Leaf	*Laurus novocanariensis* Rivas-Mart., Lousa, Fern. Prieto, E. Días, J.C. Costa & C. Aguiar	CCAC 6998 B	BEA 1758B	**PQ659168**
L 057	Tenerife	1	Leaf	*Laurus novocanariensis* Rivas-Mart., Lousa, Fern. Prieto, E. Días, J.C. Costa & C. Aguiar	CCAC 6999 B	BEA 1759B	PP409595

L 058	Tenerife	1	Leaf	*Laurus novocanariensis* Rivas-Mart., Lousa, Fern. Prieto, E. Días, J.C. Costa & C. Aguiar	CCAC 7000 B	BEA 1760B	PP409594
L 059	Tenerife	1	Leaf	*Laurus novocanariensis* Rivas-Mart., Lousa, Fern. Prieto, E. Días, J.C. Costa & C. Aguiar	CCAC 7001 B	BEA 1761B	PP409596
L 060	Tenerife	1	Leaf	*Laurus novocanariensis* Rivas-Mart., Lousa, Fern. Prieto, E. Días, J.C. Costa & C. Aguiar	CCAC 7002 B	BEA 1762B	**PQ659144**
L 061	Tenerife	1	Leaf	*Laurus novocanariensis* Rivas-Mart., Lousa, Fern. Prieto, E. Días, J.C. Costa & C. Aguiar	CCAC 7003 B	BEA 1763B	**PQ659150**
L 062	Tenerife	1	Leaf	*Laurus novocanariensis* Rivas-Mart., Lousa, Fern. Prieto, E. Días, J.C. Costa & C. Aguiar	CCAC 7004 B	BEA 1764B	**PQ659163**
L 063	Tenerife	1	Leaf	*Laurus novocanariensis* Rivas-Mart., Lousa, Fern. Prieto, E. Días, J.C. Costa & C. Aguiar	CCAC 7005 B	BEA 1765B	**PQ659163**
L 064	Tenerife	1	Leaf	*Laurus novocanariensis* Rivas-Mart., Lousa, Fern. Prieto, E. Días, J.C. Costa & C. Aguiar	CCAC 7006 B	BEA 1766B	**PQ659166**

L 065	Tenerife	1	Leaf	*Laurus novocanariensis* Rivas-Mart., Lousa, Fern. Prieto, E. Días, J.C. Costa & C. Aguiar	CCAC 7007 B	BEA 1767B	**PQ659156**
L 066	Tenerife	1	Leaf	*Laurus novocanariensis* Rivas-Mart., Lousa, Fern. Prieto, E. Días, J.C. Costa & C. Aguiar	CCAC 7008 B	BEA 1768B	**PQ659143**
L 067	Tenerife	1	Leaf	*Laurus novocanariensis* Rivas-Mart., Lousa, Fern. Prieto, E. Días, J.C. Costa & C. Aguiar	CCAC 7009 B	BEA 1769B	**PQ659143**
L 068	Tenerife	1	Leaf	*Laurus novocanariensis* Rivas-Mart., Lousa, Fern. Prieto, E. Días, J.C. Costa & C. Aguiar	CCAC 7010 B	BEA 1770B	**PQ659144**
L 069	Sao Jorge (Azores)	8	Leaf	*Laurus azoricus* (Seub.) Franco	CCAC 7011 B	BEA 1771B	**PQ659137**
L 070	Sao Jorge (Azores)	8	Leaf	*Laurus azoricus* (Seub.) Franco	CCAC 7012 B	BEA 1772B	**PQ659138**
L 071	La Palma	5	Leaf	*Ocotea foetens* (Aiton) Baill	CCAC 7013 B	BEA 1773B	**n.d.**
L 072	La Palma	5	Leaf	*Ocotea foetens* (Aiton) Baill	CCAC 7014 B	BEA 1774B	**PQ659144**
L 073	La Palma	4	Leaf	*Ocotea foetens* (Aiton) Baill	CCAC 7015 B	BEA 1775B	**PQ659140**
L 074	La Palma	4	Leaf	*Ocotea foetens* (Aiton) Baill	CCAC 7016 B	BEA 1776B	**PQ659170**
L 075	La Palma	4	Leaf	*Viburnum rugosum* Pers	CCAC 7021 B	BEA 1777B	**PQ659136**
L 076	La Palma	4	Leaf	*Viburnum rugosum* Pers	CCAC 7022 B	BEA 1778B	**PQ659172**

L 077	La Palma	4	Leaf	*Ocotea foetens* (Aiton) Baill	CCAC 7023 B	BEA 1779B	**PQ659140**
L 078	La Palma	4	Leaf	*Ocotea foetens* (Aiton) Baill	CCAC 7024 B	BEA 1780B	**PQ659161**
L 079	La Palma	5	Leaf	*Apollonia barujana* (Cav.) A. Braun	CCAC 7025 B	BEA 1781B	**PQ659133**
L 080	La Palma	5	Leaf	*Apollonia barujana* (Cav.) A. Braun	CCAC 7026 B	BEA 1782B	**PQ659153**
L 081	La Palma	5	Leaf	*Laurus novocanariensis* Rivas-Mart., Lousa, Fern. Prieto, E. Días, J.C. Costa & C. Aguiar	CCAC 7027 B	BEA 1783B	**PQ659143**
L 082	La Palma	5	Leaf	*Laurus novocanariensis* Rivas-Mart., Lousa, Fern. Prieto, E. Días, J.C. Costa & C. Aguiar	CCAC 7028 B	BEA 1784B	**PQ659158**
L 083	La Palma	5	Leaf	*Laurus novocanariensis* Rivas-Mart., Lousa, Fern. Prieto, E. Días, J.C. Costa & C. Aguiar	CCAC 7029 B	BEA 1785B	**PQ659172**
L 084	La Palma	5	Leaf	*Laurus novocanariensis* Rivas-Mart., Lousa, Fern. Prieto, E. Días, J.C. Costa & C. Aguiar	CCAC 7030 B	BEA 1786B	**PQ659160**
L 085	La Palma	4	Leaf	*Ocotea foetens* (Aiton) Baill	CCAC 7031 B	BEA 1787B	**PQ659147**
L 086	La Palma	4	Leaf	*Ocotea foetens* (Aiton) Baill	CCAC 7032 B	BEA 1788B	**PQ659135**
L 087	La Palma	4	Leaf	*Ocotea foetens* (Aiton) Baill	CCAC 7033 B	BEA 1789B	**PQ659158**

L 088	La Palma	4	Leaf	*Ocotea foetens* (Aiton) Baill	CCAC 7034 B	BEA 1790B	**PQ659159**
L 089	La Palma	3	Leaf	*Laurus novocanariensis* Rivas-Mart., Lousa, Fern. Prieto, E. Días, J.C. Costa & C. Aguiar	CCAC 7035 B	BEA 1791B	**PQ659133**
L 090	La Palma	3	Leaf	*Laurus novocanariensis* Rivas-Mart., Lousa, Fern. Prieto, E. Días, J.C. Costa & C. Aguiar	CCAC 7036 B	BEA 1792B	**PQ659134**
L 091	La Palma	4	Leaf	*Ocotea foetens* (Aiton) Baill	CCAC 7037 B	BEA 1793B	**PQ659158**
L 092	La Palma	4	Leaf	*Ocotea foetens* (Aiton) Baill	CCAC 7038 B	BEA 1794B	**PQ659158**
L 093	La Palma	5	Leaf	*Laurus novocanariensis* Rivas-Mart., Lousa, Fern. Prieto, E. Días, J.C. Costa & C. Aguiar	CCAC 7039 B	BEA 1795B	**PQ659146**
L 094	La Palma	5	Leaf	*Laurus novocanariensis* Rivas-Mart., Lousa, Fern. Prieto, E. Días, J.C. Costa & C. Aguiar	CCAC 7040 B	BEA 1796B	**PQ659146**
L 095	La Palma	5	Leaf	*Ocotea foetens* (Aiton) Baill	CCAC 7041 B	BEA 1797B	**PQ678924**
L 096	La Palma	5	Leaf	*Ocotea foetens* (Aiton) Baill	CCAC 7042 B	BEA 1798B	**PQ678924**
L 097	La Palma	5	Leaf	*Ocotea foetens* (Aiton) Baill	CCAC 7043 B	BEA 1799B	**PQ659174**
L 098	La Palma	5	Leaf	*Ocotea foetens* (Aiton) Baill	CCAC 7044 B	BEA 1800B	**PQ659174**

L 099	La Palma	5	Leaf	*Laurus novocanariensis* Rivas-Mart., Lousa, Fern. Prieto, E. Días, J.C. Costa & C. Aguiar	CCAC 7045 B	BEA 1801B	**PQ659157**
L 100	La Palma	5	Leaf	*Laurus novocanariensis* Rivas-Mart., Lousa, Fern. Prieto, E. Días, J.C. Costa & C. Aguiar	CCAC 7046 B	BEA 1802B	**PQ659157**
L 101	La Palma	5	Leaf	*Laurus novocanariensis* Rivas-Mart., Lousa, Fern. Prieto, E. Días, J.C. Costa & C. Aguiar	CCAC 7047 B	BEA 1803B	**PQ659154**
L 102	La Palma	5	Leaf	*Laurus novocanariensis* Rivas-Mart., Lousa, Fern. Prieto, E. Días, J.C. Costa & C. Aguiar	CCAC 7048 B	BEA 1804B	**PQ659154**
